# Comprehensive Evaluation of the Physicochemical Attributes, Antioxidant Capacity, and pH-Responsive Behavior of Starch Films Enhanced by Laver Incorporation

**DOI:** 10.3390/foods13111600

**Published:** 2024-05-21

**Authors:** Ying Chen, Zhu Zhu, Yunyue Ye, Qi Li, Tao Yang, Chengran Guan, Fengsong Liu

**Affiliations:** 1 School of Pharmacy, Hainan Medical University, Haikou 571199, China; 008265@yzu.edu.cn; 2 School of Food Science and Engineering, Yangzhou University, Yangzhou 225127, China; 15151159819@163.com (Z.Z.); 15896055162@163.com (Y.Y.); 15751544859@163.com (Q.L.); crguan@yzu.edu.cn (C.G.); 3 Jiangsu Dairy Biotechnology Engineering Research Center, Yangzhou 225127, China; 4 School of Food Science and Engineering, South China University of Technology, Guangzhou 510640, China

**Keywords:** starch film, laver, antioxidant, freshness indicator

## Abstract

Herein, a new starch film incorporating laver was developed to address issues related to inadequate water resistance and suboptimal preservation quality in food packaging. The integration of laver into starch film formulations offers a compelling avenue for creating biodegradable, active, and smart food packaging. Scanning electron microscope (SEM) analysis revealed that the starch film with a laver concentration of 70% exhibited a uniformly flat microstructure, as expected. Fourier-transform infrared spectroscopy (FTIR) confirmed the presence of intermolecular interactions and hydrogen bonding between the starch and laver. Viscoelastic tests demonstrated the superior film-forming performance of the starch/laver composite films. Moreover, it was found that the most favorable concentration of incorporated laver was 10%. Specifically, the S7-3 film emerged as a promising candidate for food packaging applications, boasting the highest contact angle (CA) value of 114.98 ± 1.28°, the lowest water solubility (WS) value of 15.38%, and a reduced water vapor transmission rate (WVTR) value of 2.52 g/m^2^ × h. Additionally, the S3-7 film displayed an extraordinary tensile strength of 32.47 MPa, an elongation at break of 19.04%, and a Young’s modulus of 606.83 MPa. Furthermore, the starch/laver composite films exhibited outstanding UV-blocking capabilities, exceptional pH-responsive behavior, and significant antioxidant activity, underscoring their potential for packaging applications with laver integration.

## 1. Introduction

Edible films, crafted from renewable resources such as polysaccharides, proteins, and lipids, are popular for packaging diverse food items, including meat, seafood, vegetables, fruits, and candies. These films offer improved barrier properties and antioxidant effects [1]. Researchers are exploring novel edible film materials from cereals, vegetables, and fruits. Nevertheless, numerous current films display rough surfaces and inadequate mechanical or barrier properties, underscoring the demand for new biomacromolecules with superior film-forming abilities to overcome these limitations. Although derived from natural sources, chitosan, collagen, silk protein, and cellulose possess certain drawbacks, such as inferior mechanical characteristics and vulnerability to water [2]. Conversely, starch has emerged as an attractive option for edible film production due to its cost-effectiveness, abundance, diverse botanical sources, excellent film-forming properties, and economic advantages [3]. It is widely used in papermaking, hydrogels, adhesives, and films. However, existing starch-based films face limitations such as insufficient water resistance and suboptimal food preservation capabilities, which restrict their extensive industrial application [4]. Nonetheless, starch can act as a carrier for antioxidants and antimicrobials, thus enabling the advancement of active packaging solutions. These active compounds are released into food or the package environment (e.g., headspace), thereby prolonging the storage duration of food, boosting the overall quality of the food, and enhancing the security of food [5].

Hence, researchers have begun investigating various active materials to enhance the antibacterial, antioxidant, and other functional properties of starch films, such as pectin [6], peach gum [7], sodium alginate, and others. However, most of these substances lack pH-indicating functions and cannot improve the utilization of starch films in smart active packaging. Polysaccharides, utilized in film-forming applications, offer diverse possibilities for both food and non-food uses [8]. Despite limited water vapor barrier properties, hygroscopic polysaccharides like alginate and carrageenan can form relatively thick films on food surfaces, providing temporary protection against moisture loss. Laver hydrocolloids, such as alginate and carrageenan, are intriguing in the context of biodegradable films due to their natural gel-forming properties. However, the extraction processes for these laver derivatives often involve unsustainable chemical and energy consumption. Recent research indicates that raw laver can create an edible film with functional attributes, but its inherent hydrophilicity results in subpar mechanical and water barrier properties [9]. As a result, modification through grafting/blending with other polymers or incorporating fillers is often necessary to compete with conventional polymers. Laver-based polysaccharides are abundant biopolymers with utility across various industries, serving as scaffolds, dispersants, coatings, stabilizing agents, and packaging materials in food, biomedical, and packaging applications. Laver, a widely cultivated and commercially available seaweed, is renowned for its appealing flavor and rich nutritional components, including lipids, protein, vitamins, and minerals. On the other hand, microalgae contain biologically active compounds with antioxidant, anti-inflammatory, and coloring effects, and are already marketed as food supplements, while also being used to enhance the nutritional content of traditional foods [10]. Furthermore, including chemically active molecules in film formulations is a promising strategy for enhancing the functional properties of films and reducing reliance on synthetic chemicals, especially in food packaging and pharmaceutical applications. Laver, with its high content of phenolic compounds and anthocyanins, holds substantial promise for actively contributing to film functionality [11]. Anthocyanins are known for their ability to change color under different pH conditions, making them suitable for use in specific food products that exhibit pH variations. Integrating laver into film formulations presents an exciting opportunity for producing biodegradable, active, and intelligent food packaging. This approach not only increases the value of laver waste but also reduces waste generation by fostering the creation of eco-friendly products.

The aim of this study was to formulate and characterize a new starch film with laver for food packaging. Various laver contents were incorporated into starch film formulations to assess their impact on film performance. As hypothesized, the intermolecular hydrogen bonding between starch and laver could potentially improve the water resistance of the starch films. In particular, several moisture sensitivity parameters, such as contact angle (CA), water vapor transmission rate (WVTR), and water solubility (WS) were thoroughly investigated. Scanning electron microscopy (SEM) was employed to observe the surface and cross-sectional morphologies of the films, while Fourier-transform infrared reflection (FTIR) was utilized to evaluate potential intermolecular interactions. Additionally, the viscosity of the starch solutions was measured. Moreover, the light transmittance of the films was examined using an ultraviolet-visible (UV-vis) spectrophotometer. The mechanical properties, encompassing elongation at break, tensile strength, and Young’s modulus were comprehensively analyzed. The antioxidant activity and pH responsiveness were characterized for fresh indication and food packaging applications. The primary research objective was to establish both theoretical foundations and practical guidelines for the development of a pH-responsive packaging film, while simultaneously addressing its inadequate hydrophobicity and mechanical properties, thereby facilitating its broader utilization in food packaging applications.

## 2. Materials and Methods

### 2.1. Materials

The hydroxypropyl high-amylose corn starch used in this study was obtained from Penford, Melbourne, Australia. Its moisture content ranged from 7% to 9%, with an amylose content of 80%. The plasticizer, glycerol (AR, 99%), was supplied by Guangzhou Macklin Biotechnology Co., Ltd., Guangzhou, China. Laver was sourced from a local market on Wushan Road in Guangzhou, China, and dehydrated at 60 °C for approximately 6 h in an electric thermostatic drying oven (DNF610, YAMATO Scientific Co., Ltd., Tokyo, Japan). After dehydration, the laver was pulverized and sifted through a No. 100 mesh. Subsequently, 200 g of powder was subjected to extraction using 10 L of deionized water with stirring for 5 h at 90 °C. The resulting extract was obtained by centrifuging at 5000 rpm for 5 min. The supernatant was then mixed and concentrated to one-tenth of the original volume at reduced pressure, and subsequently lyophilized to produce a laver extract.

### 2.2. Preparation of Starch/Laver Films

**Scheme 1:**
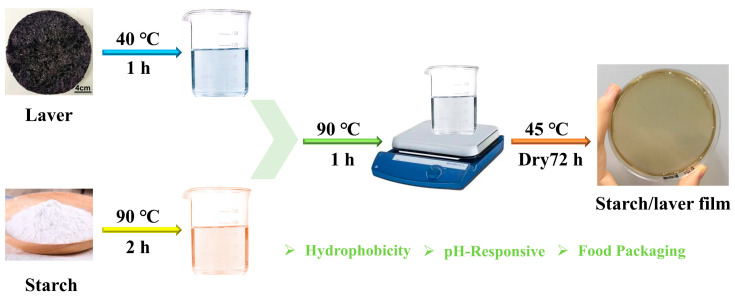
Diagram of the fabrication of starch/laver films.

To prepare the starch films, an optimized method for solution casting was implemented. The experimental procedure began with the generation of a gelatinized corn starch solution. Creating a fully plasticized starch solution involved carefully blending 5 g of starch, 1 g of glycerol, and 100 mL of deionized water at 90 °C for 2 h. Subsequently, film-forming dispersions were generated using a 4% polymer suspension (*w*/*w*) with varying S:LE proportions of 0:100, 30:70, 50:50, 70:30, and 100:0. Initially, separate dispersions of starch and laver extract were fabricated. The starch aqueous dispersions underwent gelatinization at 90 °C for 60 min. Following this, laver extraction was introduced to the dispersions, which were mixed using magnetic stirring before being homogenized with a rotor-stator homogenizer (Ultraturrax D125, Janke and Kunkel, Karlsruhe, Germany) at 2100 rpm for 1 h. In a three-necked flask, a 4% (*w*/*w*) starch solution in water was prepared and gradually heated from ambient temperature to 90 °C while continuously stirring for 1 h to achieve starch gelatinization. The starch solution and obtained composite suspensions were immediately transferred to polyethylene plates and left undisturbed in an oven at 45 °C for 72 h, as shown in Scheme 1. The cast films achieved a final thickness of approximately 0.10 mm, which was regulated by determining the amount of starch suspension poured onto the plate, and measured using a micrometer (µm). Prior to additional testing, all fabricated films underwent conditioning in a sodium bromide saturated environment for 24 h at 57% and 75% relative humidity (RH).

### 2.3. Characterization of Film Properties

#### 2.3.1. SEM Morphology of the Starch Films

For observing the surface and interface morphologies of the starch films, an SEM instrument (ZEISS, Oberkochen, Germany) operating at a lower voltage of 5 kV was utilized to prevent damage to the surfaces. The samples were frozen using liquid nitrogen and manually fractured to capture images of the top surfaces and cross-sections. Prior to imaging, each film was mounted onto a double-sided adhesive metal stub and then gold plated using an Eiko sputter coater for 90 s under vacuum spraying to guarantee conductivity.

#### 2.3.2. Fourier-Transform Infrared Spectroscopy (FTIR)

To explore potential interactions between starch and laver, a Tensor spectrophotometer (Invenio, Bruker, Ettlingen, Germany) was employed. FTIR spectra of the films were measured using the attenuated total reflectance (ATR) method. The ATR-FTIR spectra, comprising 64 scans, spanned a spectral range of 600–4000 cm^−1^ with a resolution of 4 cm^−1^ in each case [12].

#### 2.3.3. Viscosity Measurements

The viscosity of different starch solutions was assessed at ambient temperature utilizing a Rheometer (HR-2 Discovery Hybrid Rheometer, TA Instruments, New Castle, DE, USA) set to a constant rotational speed of 30 rpm for all samples. Temperature stability was maintained through a Peltier system, with platinum resistance thermometer sensors (accuracy to ±0.1 °C) continuously monitoring the temperature [13]. To ensure a thermal balance between the solution and the spindle while maintaining shear, the viscometer spindle was submerged in the solution for approximately 3 min. Each test was conducted in triplicate for accuracy and consistency.

#### 2.3.4. Contact Angle (CA)

The surface hydrophobicity of the starch films was examined by measuring the change in CA when a water droplet was placed on their surface using a Data Physics contact angle goniometer (model OCA 20, Data Physics, Stuttgart, Germany) via the sessile-drop method [14]. Three droplets were used for each sample to obtain an average CA value. Before testing, all of the samples tested were subjected to 57% RH for 10 h.

#### 2.3.5. Water Solubility (WS)

To ascertain the initial dry mass of the starch films, they were transformed into circular discs with a 20 mm radius and subsequently dried in an oven at 70 °C for 2 h. Subsequently, the films were placed in capped falcon tubes (50 mL) with 25 mL of distilled water. These tubes were then moved to a shaker oven (Jal Tajhiz Labtech. Co., Ltd., Tehran, Iran) at 25 °C for 24 h. The purpose of this step was to allow the films to dissolve in water. Following the immersion period, the solution was filtrated through Whatman filter paper to collect any remaining undissolved film fragments. The remaining fragments were dried again in the oven at 70 °C for 2 h and weighed to determine the final dry mass of the film. The WS was assessed by calculating the percentage of weight loss, indicating the film’s dissolution in water [15]. To ensure precision, each film sample was measured at least five times, and the average value was calculated.

#### 2.3.6. Water Vapor Transmission Rate (WVTR)

To assess the WVTR of the starch films, we adapted a methodology similar to that outlined by Liu et al., incorporating some modifications [16]. The experiments were conducted in triplicate using a thermos hygrometer and deionized water, following the standard testing procedure ASTM E96/E96M-14 (water vapor permeability tester, WVTR-2501, SYSTESTER, Jinan, China). For measurement, specialized cups with an average diameter of 4 cm and a depth of 2.5 cm were employed. The films were cut into discs slightly larger than the cup diameter and positioned over each cup, which was filled with 5 g of distilled water. To create a desiccation environment, the cups were positioned in a desiccator containing sufficient anhydrous CaCl_2_. The increase in weight due to water vapor transport was monitored by weighing the cups at 24 h intervals. The WVTR was then calculated by dividing the slope (g/h) by the transfer area (m^2^).

#### 2.3.7. Transparency of the Film

To determine the film transparency, an Agilent 8453 UV-Vis spectrophotometer was utilized (Suzhou, China). Films, cut to dimensions of 4 cm in length and 2 cm in width, were firmly affixed to the measuring cell using adhesive tape. The transmittance spectrum, spanning from 200 to 780 nm, was recorded, with the transmittance at 600 nm utilized for transparency calculations [17]. To enhance the precision and mitigate potential measurement discrepancies, each film sample was subjected to a minimum of three measurements. Furthermore, the film transparency (T) was calculated using the following equation.

T = (−*log T*_600_)/*L* (1)

In this equation, T is the transparency value, while *T*_600_ and *L* are the optical transmittance of the film at 600 nm and the film thickness, respectively.

#### 2.3.8. Tensile Properties

The assessment of tensile characteristics, including tensile strength, Young’s modulus, and elongation at break, adhered to the guidelines specified in ASTM D882-12. Bar-shaped tensile specimens were derived by cutting from the casting films to meet specific dimensions. Tensile testing was conducted using the Instron 5565 apparatus sourced from the United States (Instron, Norwood, MA, USA), operating at a crosshead speed of 5 mm/min under ambient temperature conditions (25 °C). Prior to testing, all specimens underwent a conditioning period of 24 h at relative humidity levels of 57% and 75%. To ensure reproducibility, a minimum of seven film samples were subjected to testing under each condition [18].

#### 2.3.9. Color Difference

To ascertain the color response of various sample solutions, a color difference analyzer (Ci 7800, X-rite, Grand Rapids, MI, USA) was employed [19]. To mitigate experimental error, each sample was averaged over at least five measurements. The following equations were utilized to determine the values of ΔE and WI:

∆E = [(L* − L) 2 + (a* − a)2 + (b* − b)2]1/2(2)

WI = 100 − [(100 − L)2 + a2 + b2]1/2(3)

#### 2.3.10. Determination of Antioxidant Activity

The films’ free-radical scavenging capacity was analyzed using the 1,1-diphenyl-2-picrylhydrazyl (DPPH) test. A DPPH solution was prepared by dissolving 5 mg of DPPH in 100 mL of ethanol [20]. For the sample solution, 0.5 g of film was dissolved in distilled water (10 mg/mL) and underwent 1 h ultrasonic treatment for extraction, followed by an additional 24 h extraction. After centrifugation to separate the supernatant from solid particles, the DPPH solution and the extracted sample solution were mixed at a 1:1 ratio. The mixed solution stood undisturbed for 30 min, and the absorbance was measured at a wavelength of 517 nm. The DPPH radical scavenging activity was evaluated using the following equation:

Scavenging activity (%) = (*A*_0_ − *A_i_*)/*A*_0_ × 100% (4)

where, *A*_0_ refers to the absorbance of the control solution, and *A_i_* represents the absorbance of the sample solution mixed with the DPPH solution.

### 2.4. Statistical Analysis

For the analysis and generation of figures in this study, Chemical Draw (Ultra 14.0) and Origin Pro (version 8.0) software by Stat-Ease Inc. in Minneapolis, MN, USA were employed. Experimental data were expressed as mean values ± standard deviations (SD). Statistically significant differences (*p* < 0.05) were identified using the Statistical Program for Social Sciences (SPSS) version 22.0.

## 3. Results and Discussion

### 3.1. SEM Morphology Analysis of the Starch Films

**Figure 1:**
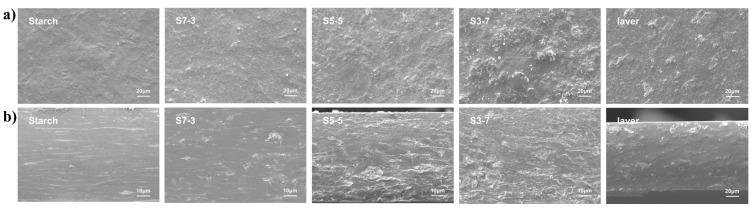
SEM pictures of the starch-based films. (**a**): Surface and (**b**): cross-section.

The observation of the starch-based films’ top surface and cross-section (Figure 1) aimed to assess their morphologies and the starch-laver compatibility. The pure starch film exhibited a remarkably smooth and flat surface, whereas the pure laver film showed a rough microstructure, indicating an inferior film-forming performance. Adding laver to the starch film resulted in an uneven structure with distinct fragments on the surfaces of the S7-3, S5-5, and S3-7 films, reflecting incompatibility. However, incorporating laver facilitated the creation of an initially homogeneous and later rough surface structure due to intermolecular interactions between starch and laver. Cross-section analysis revealed that the starch film had a uniform structure, while the pure laver film displayed a rough microstructure. Integrating laver into the starch films resulted in rough cross-section structures. With an increase in laver concentration, both the surface and cross-sectional morphologies displayed decreased uniformity. The S3-7 film, with a 70% laver concentration, demonstrated a relatively homogeneous and flat structure, indicating an exceptional film-forming capability and a superior performance.

### 3.2. Chemical Interactions between Starch and Laver

**Figure 2:**
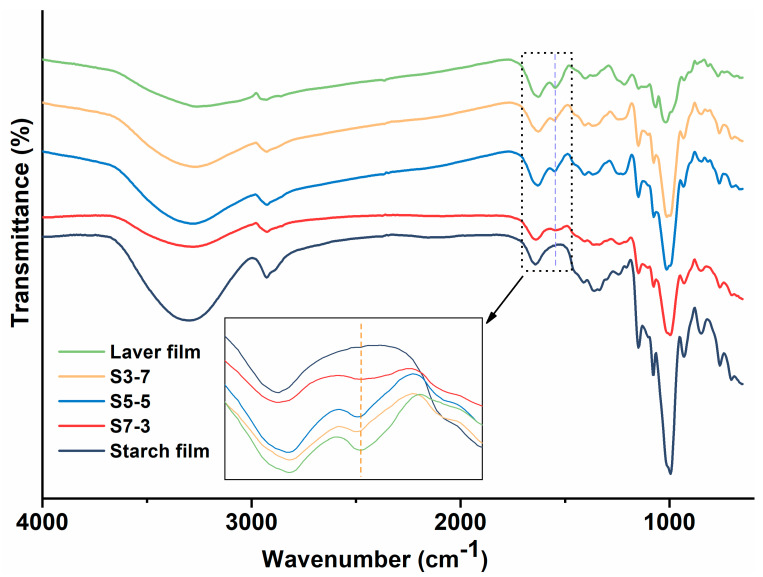
FTIR spectra of the starch-based films.

Figure 2 displays the FTIR spectra of different film types: starch film, S7-3 film, S5-5 film, S3-7 film, and pure laver film. In all film samples, a substantial absorption peak was observed at 3281 cm^−1^, attributable to the O-H stretching vibration found in both the starch and laver spectra. The incorporation of laver into the starch film led to a significant decrease in the intensity of the broad O-H band, ascribed to strong intermolecular interactions that diminish the -OH groups in starch. Additionally, all films displayed stretching peaks at 2928 cm^−1^, indicative of C-H bending vibrations, and absorption peaks at 1637 cm^−1^, corresponding to H-O-H bending vibrations in starch and laver. The FTIR spectra of the S7-3 film, S5-5 film, S3-7 film, and pure laver film revealed a distinct absorption peak at 1546 cm^−1^, representing the stretching vibrations of laver. Intermolecular hydrogen bonding in the hybrid composites indicated good compatibility between starch and laver matrices. Effective intermolecular interactions and hydrogen bonding contributed to a significant reduction in -OH groups, potentially enhancing the moisture repellency and gas permeability of the starch-based films. Thorough hydrophobic characterizations are necessary to confirm this improvement.

### 3.3. Viscoelastic Properties of the Starch-Based Films

**Figure 3:**
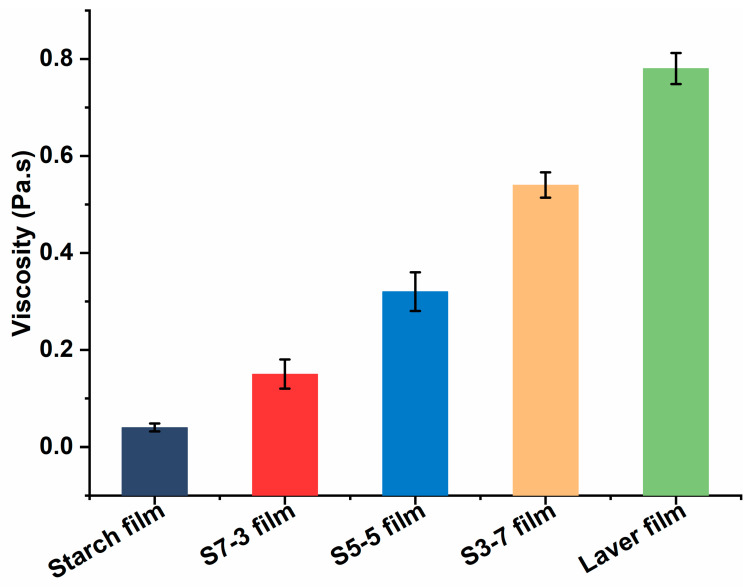
Viscosity of the starch films.

Viscosity is a crucial parameter indicating a fluid’s resistance to shape changes or relative motion between adjacent parts, and plays a pivotal role in the film-forming performance. As illustrated in Figure 3, pure starch demonstrated significantly lower viscosity compared to laver film, primarily attributed to the robust gelation properties of laver. However, with the addition of laver to the starch film, there is a pronounced increase in the viscosity of the starch. Specifically, the S7-3 film, S5-5 film, S3-7 film, and pure laver film exhibited viscosities of 0.15, 0.32, 0.54, and 0.78 Pa·s, respectively. This heightened viscosity can be ascribed to the unique proteins and carbohydrates in laver that intertwine with the starch molecular chains [21]. This interaction facilitates polymer chain movement and enhances the sol-gel properties of the blend. Therefore, it is reasonable to assume that the starch/laver composite films exhibit exceptional film-forming performance, particularly when the laver addition reaches 70%.

### 3.4. Hydrophobicity of the Starch-Based Films

**Figure 4:**
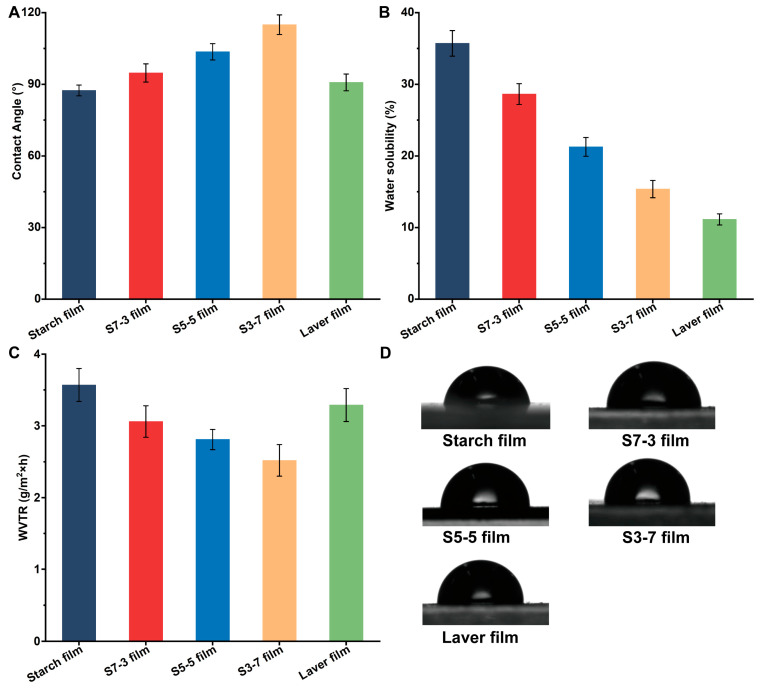
Hydrophobicity of the starch films. (**A**): CA; (**B**): WS; (**C**): WVTR; and (**D**): optical images.

In order to comprehensively assess the overall hydrophobic properties of the starch films, various characteristics such as WS, CA, and WVTR were investigated (Figure 4). Figure 4A,D illustrate the surface wettability of the starch-based films and the optical images of CA. Notably, the pure starch film exhibited a minimum CA value of 87.45 ± 1.36°, signifying a substantial hydrophilicity compared to the remaining films. With the addition of laver, a significant increase was observed in the CA values for the S7-3 film, S5-5 film, and S3-7 film, which reached 94.77 ± 1.51°, 103.63 ± 1.45°, and 114.98 ± 1.28°, respectively. This enhancement is due to the establishment of hydrogen bonding between starch and laver, which efficiently improves the surface water resistance of the starch films [22]. Specifically, the CA value of the S7-3 film showed a notable elevation of 24.16 ± 0.57° compared to the laver film, highlighting the successful modification of the surface to achieve a correspondingly hydrophobic nature through laver integration. Based on the data in Figure 4B, the starch film demonstrated the highest moisture sensitivity, with a WS value of 35.72%. Remarkably, the S7-3 film, S5-5 film, and S3-7 film displayed reduced water solubility. This observed trend can account for the intermolecular entanglement and hydrogen bonding between starch and laver, crucial in mitigating the water sensitivity and forming a denser surface. The natural gum components in laver may form a cross-linked network with starch, decreasing the solubility of the starch in water. The laver film showed the lowest WS at 11.15%, likely due to laver being derived from seaweed extracts with low water solubility. Specifically, the S7-3 film had the lowest WS at 15.38%. In summary, the introduction of laver decreased the water solubility of the starch-based films, as indicated by the results mentioned above. The WVTR of various films, including the starch film, S7-3 film, S5-5 film, S3-7 film and laver film, was assessed for their water vapor permeability, as depicted in Figure 4C. According to the results, the starch film exhibited the greatest WVTR values of 3.57 g/m^2^ × h, attributable to the large number of hydroxyl groups in the side chain of the starch molecule, causing weak water permeability and increased moisture susceptibility [23]. Additionally, the WVTR values of the laver film were similar to those of the starch films, demonstrating restricted water vapor permeability. Interestingly, significant decreases in WVTR were noted in the S7-3 film, S5-5 film, and S3-7 film, with values of 3.06 g/m^2^ × h, 2.81 g/m^2^ × h, and 2.52 g/m^2^ × h, respectively. This reduction was primarily due to the chemical intertwinement and intermolecular interactions between the starch and laver, enhancing the water vapor permeability of the starch-based films [24]. It is noteworthy that the laver film revealed the lowest permeability to water vapor, evident from its WVTR value of 3.29 g/m^2^ × h.

WAC, CA, and WVTR are critical parameters influencing the surface hydrophobicity and water vapor permeability of starch films, crucial for determining the quality and shelf-life of food packaging [16,25,26]. Hence, understanding and controlling these parameters in starch films is essential for achieving the desired surface water resistance and gas permeability, ultimately impacting the overall efficacy of food packaging and extending the shelf life of packaged food products. Of the S7-3 film, the S5-5 film and the S3-7 film, the S7-3 film appears to have the greatest potential for use in food packaging applications. It achieved the highest CA value, ranging from 94.77 ± 1.51° to 114.98 ± 1.28°, the lowest WS value, decreasing from 28.65% to 15.38%, and the lowest WVTR value, dropping from 3.06 g/m^2^ × h to 2.52 g/m^2^ × h.

### 3.5. Transparency of the Starch-Based Films

**Figure 5:**
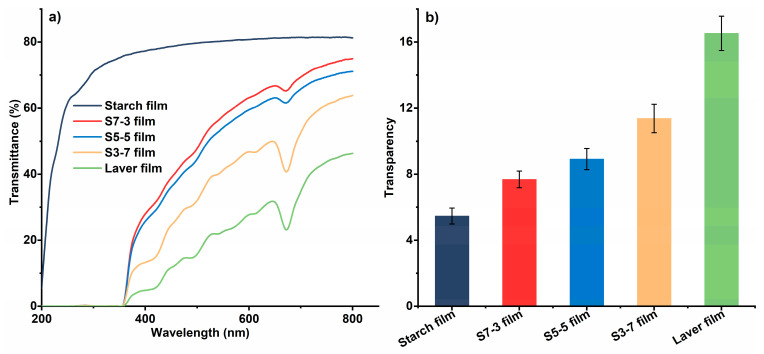
Optical transmittance of the starch-based films. (**a**): UV-vis spectra and (**b**): transparency.

Figure 5a shows the UV-Vis spectra of the starch-based films subjected to ultraviolet (UV) light (200–400 nm) and visible (vis) light (400–780 nm). In general, films with superior UV-blocking capabilities and higher light transmittance are promising for packaging applications. With the incorporation of laver, the S3-7 film showed the lowest transmittance in the UV range, indicating superior UV-blocking properties. This observed phenomenon may be due to the presence of pigments and polysaccharides in laver, which have inherent UV absorption capabilities. These components reduce the penetration of UV light, thereby enhancing the UV-blocking performance of the film. It was noted that the starch film displayed the exceptional optical transmittance of 81.26%, indicating a uniform structure. A subsequent reduction in light transmittance was noticed after increasing the concentration of laver. The S7-3 film, S5-5 film, and S3-7 film had maximum light transmittances of 74.99%, 71.13%, and 63.87%, respectively. Notably, all modified films showed enhanced UV-blocking performance with laver. According to Figure 5b, the optical transparency of the starch film was measured at 5.47, indicating a high level of light transmittance. Additionally, the S7-3 film, S5-5 film, and S3-7 film demonstrated optical transparency values of 7.69, 8.92, and 11.38, respectively. Specifically, all the modified starch films showcased exceptional UV-blocking performance, highlighting their potential for packaging applications with laver integration [27].

### 3.6. Tensile Performance of the Starch Films

**Figure 6:**
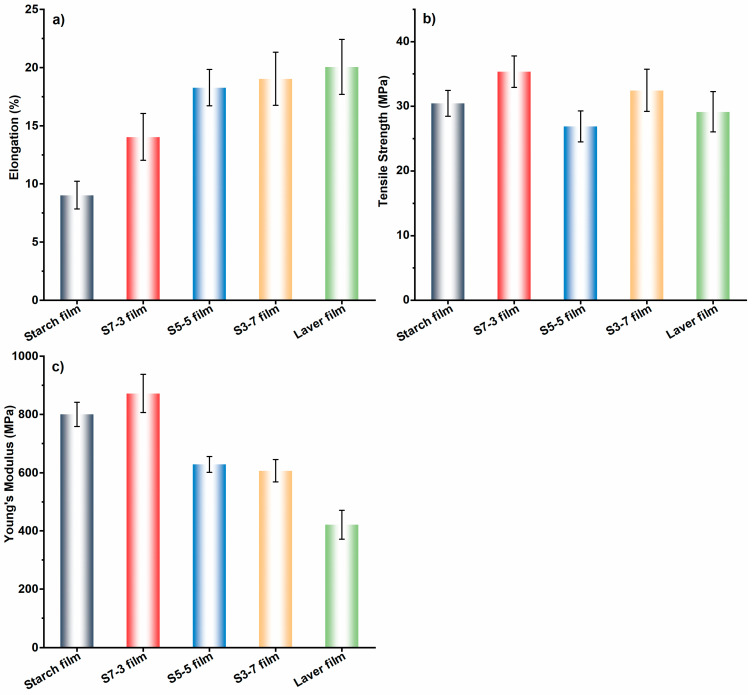
Mechanical characteristics of the starch-based films. (**a**): Elongation at break; (**b**): tensile strength; and (**c**): Young’s modulus.

The mechanical properties of the starch film, S7-3 film, S5-5 film, S3-7 film, and laver film were evaluated for practical applications, as shown in Figure 6. Distinct trends in the elongation at break, tensile strength, and Young’s modulus were evident with varying laver concentrations. The elongation value for the starch film was 9.03%. Upon incorporating laver, the S7-3 film, S5-5 film, and S3-7 film showed an increased elongation at break of 14.04%, 18.27%, and 19.04%, respectively. This enhancement is caused by the interactions and hydrogen bonds between the laver and starch chains, which facilitate the formation of a dense and compact film structure, thereby significantly enhancing both the film rigidity and ductility [28]. As observed in Figure 6b, the tensile strength of the S7-3 film, S5-5 film, and S3-7 film were 35.37 MPa, 26.92 MPa, and 32.47 MPa, respectively, indicating excellent compatibility between laver and starch. Furthermore, the Young’s moduli of the S7-3 film, S5-5 film, and S3-7 film were 871.66 MPa, 628.54 MPa, and 606.83 MPa, respectively. In conclusion, the S3-7 film exhibited an exceptional elongation at break of 19.04%, a tensile strength of 32.47 MPa, and a Young’s modulus of 606.83 MPa, showcasing a high potential for food packaging applications involving starch/laver composite films.

### 3.7. pH Responsive Performance

**Figure 7:**
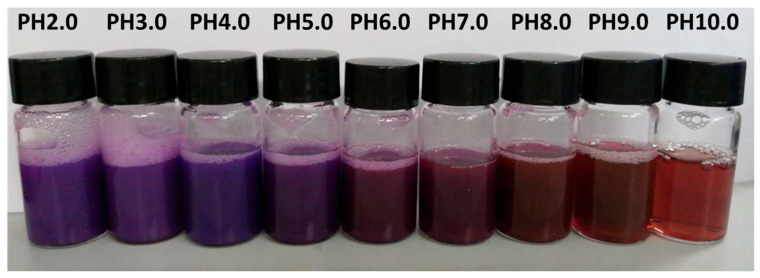
pH-responsive properties of laver extraction.

Table 1 displays the color difference values for the starch film, S7-3 film, S5-5 film, S3-7 film, and laver film. It is noteworthy that the starch film demonstrated the highest L value of 94.45, signifying high transparency. The L values of the S7-3 film, S5-5 film, and S3-7 film decreased with laver addition, owing to the absorption of the specific laver components’ wavelengths of light, consistent with prior light transmittance test outcomes. Furthermore, the ∆E value displayed a substantial increasing trend with the escalating laver content. Figure 7 depicts the pH-sensitive performance of laver extraction. The pH indicator, which is widely recognized for the real-time monitoring of the freshness of food products, holds considerable promise for application in the food packaging sector [29,30,31]. The color of the laver extract gradually changed from purple to red as the pH increased, showcasing robust pH-sensitive properties. Thus, the starch/laver composite film is deemed highly applicable for freshness indication and food preservation.

### 3.8. Antioxidant Activity

**Figure 8:**
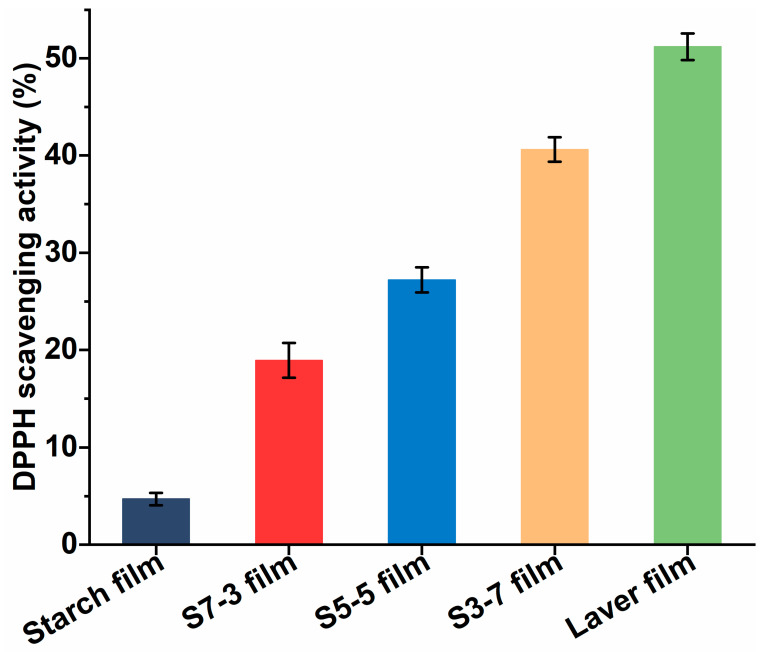
DPPH scavenging activity of the films.

In addition to the hydrophobicity and gas permeability, antioxidant activity is a crucial factor in food preservation [32]. The antioxidant efficacy of starch films, evaluated through DDPH scavenging activity, is depicted in Figure 8. Notably, the DDPH scavenging activity of the starch film was only 4.69%, suggesting a lack of antioxidant activity. A substantial difference is evident between starch films with and without laver. Furthermore, all composite films exhibit a clear increasing trend in antioxidant activity with the rise in laver concentration. The free-radical scavenging activity of the S7-3 film, S5-5 film, and S3-7 film was 18.93%, 27.21%, and 40.62%, respectively. This enhancement is attributed to the laver’s richness in antioxidants [33,34,35,36]. Figure 8 highlights that the laver film demonstrates excellent antioxidant properties, boasting a radical scavenging activity of 51.18%, nearly twice that of the S5-5 film. In summary, the integration of laver into the starch film is a promising candidate for food packaging applications [37].

## 4. Conclusions

In this study, a new starch film was fabricated by integrating laver to address concerns regarding insufficient water resistance and poor preservation quality in food packaging. The addition of laver into starch film formulations is proposed as an effective strategy for producing biodegradable, active, and intelligent food packaging. The introduction of laver facilitated the creation of a dense microstructure in the starch films, characterized by intermolecular hydrogen bonding between laver and starch, as validated by SEM and FTIR analyses. Viscoelastic tests affirmed anticipated enhancements in film formation and compatibility, leading to the formation of a more even and dense structure. Notably, the S7-3 film exhibited promising characteristics for food packaging applications, with the highest CA value, 114.98 ± 1.28°, the lowest WS value, 15.38%, and the lowest WVTR value, 2.52 g/m^2^ × h. Based on these results, the most favorable concentration of incorporated laver was found to be 10%. Furthermore, the starch/laver composite films demonstrated pH-responsive behavior and significant antioxidant activity, underscoring their potential for packaging applications. These promising outcomes highlight the considerable promise of starch films for a wide array of packaging opportunities. The incorporation of 10% laver efficiently restricted the flexibility of the starch chains, leading to the development of a compact network structure with pronounced hydrogen bonds and intermolecular entanglements. Specifically, the S3-7 film displayed the exceptional tensile strength of 32.47 MPa, an elongation at break of 19.04%, and a Young’s modulus of 606.83 MPa, showcasing superior UV-blocking capabilities. Consequently, this research is anticipated to broaden the scope of applicability of starch films to incorporate a wide range of packaging applications.

## Figures and Tables

**Table 1:** L, a, b, ∆E, and WI values of the starch films.

Sample	L	a	b	∆E	WI
Starch film	94.45 ± 0.26 ^a^	0.27 ± 0.17 ^e^	0.20 ± 0.95 ^e^	1.34 ± 0.85 ^e^	94.29 ± 0.06 ^a^
S7-3 film	86.6 ± 1.66 ^b^	1.59 ± 0.64 ^d^	13.99 ± 3.85 ^d^	17.37 ± 4.20 ^d^	80.49 ± 3.91 ^b^
S5-5 film	63.57 ± 2.29 ^d^	7.74 ± 0.56 ^c^	33.74 ± 0.25 ^c^	47.69 ± 1.79 ^c^	49.73 ± 1.91 ^c^
S3-7 film	70.74 ± 1.81 ^c^	10.52 ± 0.19 ^b^	43.01 ± 1.59 ^a^	51.57 ± 2.40 ^b^	46.92 ± 2.45 ^c^
Laver film	57.38 ± 3.86 ^d^	12.52 ± 0.22 ^a^	38.36 ± 0.77 ^b^	56.15 ± 0.85 ^a^	41.29 ± 0.96 ^d^

Values are presented as mean ± standard deviation. Statistical significance (*p* < 0.05) is indicated by different subscripts from a–e.

## Data Availability

The original contributions presented in the study are included in the article, further inquiries can be directed to the corresponding author.

## Abbreviations

SEM, scanning electron microscopy; FTIR, Fourier-transform infrared spectroscopy; CA, contact angle; WS, water solubility; WVTR, water vapor transmission rate; UV-vis, ultraviolet-visible; RH, relative humidity; ATR, attenuated total reflectance; DPPH, 1,1-diphenyl-2-picrylhydrazyl; SD, standard deviations; Spass, Statistical Program for Social Sciences.
